# Correction: The role of oscillations in grid cells’ toroidal topology

**DOI:** 10.1371/journal.pcbi.1013623

**Published:** 2025-10-29

**Authors:** Giovanni di Sarra, Siddharth Jha, Yasser Roudi

In Fig 2, two of the panels of Fig 2B are duplicates of panel Fig 2A. Please see the correct Fig 2 here.

**Fig 2:**
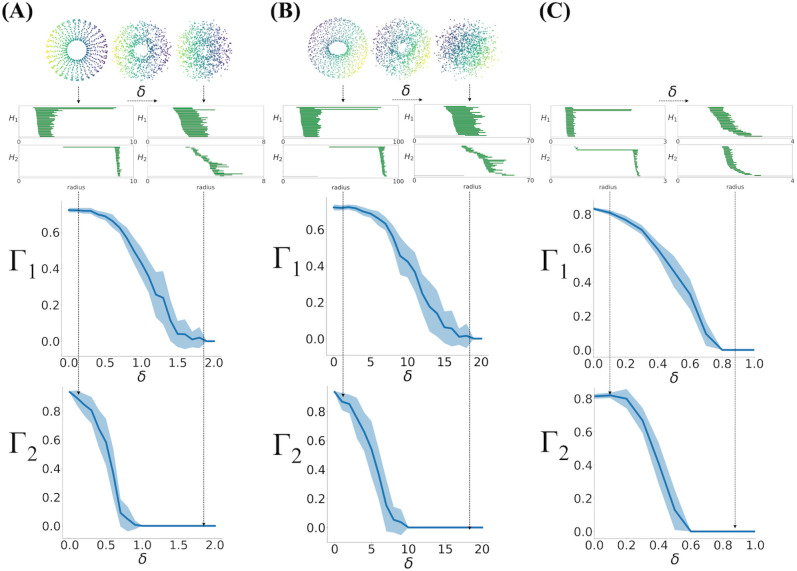
Sigmoidal relationship between Γ and noise added to simulated tori. **(A)** Zero mean Gaussian noise with standard deviation δ was added to 1200 points on a 3-dimensional torus defined by Eq. (2) with a = 5 and c = 10, shown on the top row for three increasing values of δ. Persistent homology is performed on this dataset. Barcodes for low (left) and high (right) noise levels are shown in the middle row. The two plots on the bottom show the components of the degree of toroidality, **Γ**= (Γ1, Γ2) versus the size of the noise, δ. The solid line shows the average over 20 realizations of the noise, and the shaded region is the standard deviation. The vertical arrows connects the torus, barcodes and toroidality for two values of δ. **(B)** Same as (A) with a = 50 and c = 100. **(C)** Same as (A, B) for 20 realizations of data generated on a 6-dimensional torus from Eq (9), in Methods.

## References

[1] di Sarra G, Jha S, Roudi Y. The role of oscillations in grid cells’ toroidal topology. PLoS Comput Biol. 2025;21(1):e1012776. doi: 10.1371/journal.pcbi.1012776 39879234 PMC12165393

