# Supplementary material for: The role of oscillations in grid cells’ toroidal topology
Source: PLoS Comput Biol. 2025 Jan 29;21(1):e1012776. doi: 10.1371/journal.pcbi.1012776 (PMC12165393; doi:10.1371/journal.pcbi.1012776)
Supplement: S1 Appendix — (PDF) [file pcbi.1012776.s001.pdf]

S1 Appendix – Persistent homology and the selection of time points and population vectors

The results described in the main text as well as those of [12] are found taking population vectors at all times binned at 10 ms and applying persistent homology on a subset of them. This subset, however, is not a random subset. First, only one every fifth time bin is retained in the analysis, and then the population vectors are sorted according to their mean activity, selecting the 15000 most active vectors for persistence homology. Although, in general, downsampling is justified for computational reasons, as we will show here, the particular choice of the *high activity downsampling* has a drastic effect on the results [30-32], and reinforces the conclusions that in experimental data, oscillations play a central role in the emergence of toroidal topology.

In real data, a random downsampling, that is, randomly choosing the time points, instead of high activity downsampling, severely hampers the detection of toroidal topology. This is shown in Fig A1 for two example modules; the remaining modules are shown Fig A5.

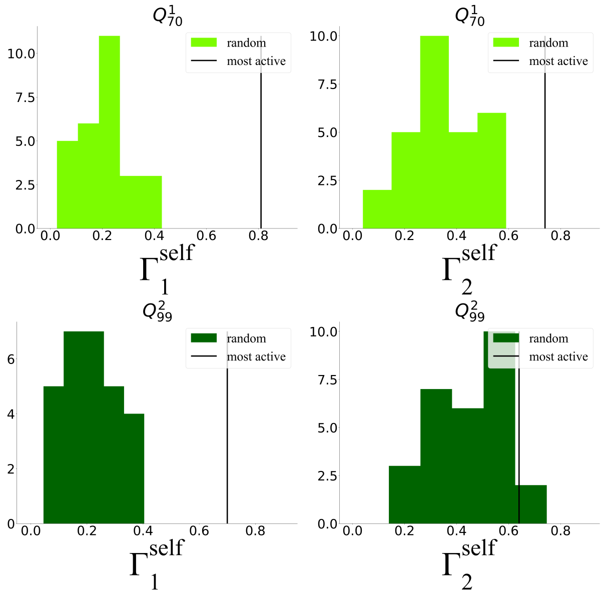

Figure A1: **Persistent homology and the selection of time points in real data.** Each histogram is a set of 30 different random selections of 15000 times for downsampling for modules Q^1_{79} and Q^2_{99}. The vertical black line shows the single realization were time points are chosen to be the 15000 ones with highest population vector activity.

Similarly, for simulations with oscillations – where high activity downsampling leads to toroidal topology– a detrimental effect on the degree of toroidality

is observed when random downsampling is performed; see Fig A2.

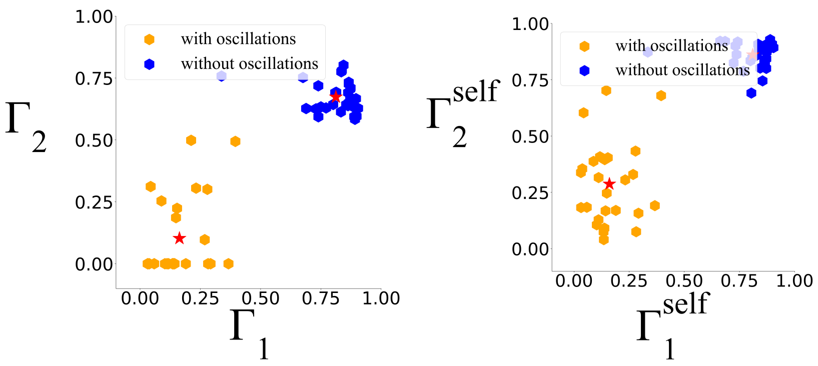

Figure A2: **Persistent homology and the selection of time points in simulations.** Scatter plots of Γ_1 vs Γ_2 and Γ_1^{self} vs Γ_2^{self} show that oscillations decrease toroidality in simulations when spike trains are downsampled randomly. The mean is shown as a red point and the values with standard deviation are the following: $\Gamma_1 = 0.16 \pm 0.01$, $\Gamma_2 = 0.10 \pm 0.16$, $\Gamma_1^{self} = 0.16 \pm 0.01$ and $\Gamma_2^{self} = 0.29 \pm 0.18$ in the case with oscillations, and $\Gamma_1 = 0.81 \pm 0.11$, $\Gamma_2 = 0.67 \pm 0.06$, $\Gamma_1^{self} = 81 \pm 0.11$ and $\Gamma_2^{self} = 86 \pm 0.06$ in the case without oscillations.

On the other hand, data from Poisson simulations in the absence of oscillations exhibit the opposite pattern. In this case, as discussed in the main text, the high activity downsampling that leads to toroidal topology in real data and simulations with oscillations, does not yield barcodes similar to data: even when the long bar in H_2 is present, it appears at scales that the bars in H_1 have disappeared. However, similar to the results of [14] (who used a geometric downsampling) , they may show consistent barcodes when time points are chosen randomly; see Fig A2. These differences between the real data and Poisson simulations with oscillations on the one hand, and Poisson simulations in the absence of oscillations on the other, is not only a consequence of mean firing rate of the neurons. In fact, as shown in Figs 10C and 10D of the main text, Figs A3 and S7, increasing mean firing rate alone, which is controlled by the parameter G_0 in the simulations, does not have an effect on the difference that oscillations cause in simulations. Moreover, jittering simulated data exhibits a similar trend to the experimental data and it doesn't change with G_0; see Fig A4.

It thus appears that the effect is caused by how the high firing rate and oscillations interact in forming the correlations necessary for the detection of toroidal topology. In other words, the temporal correlations between spike trains that lead to toroidal topology in the real data are not limited to those arising from the overlap between grid fields, which are also captured by Poisson spiking, but also involve the oscillatory components. In real data and simulations, population vectors at some time points may have higher mean activity than other time points. In the Poisson spiking network without oscillations this is due to

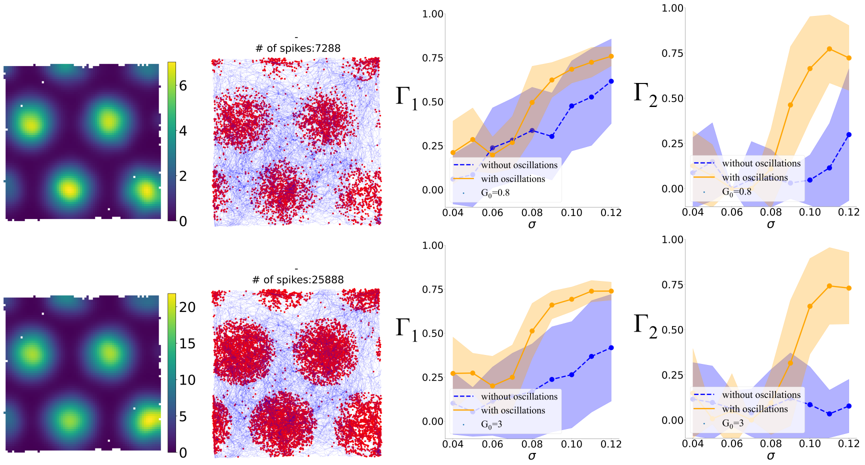

Figure A3: **Dependence of Γ on firing rate and σ.** Behavior of Γ_1 and Γ_2 in the cases with and without oscillations as in Fig 8, for two values of G_0: $G_0 = 0.8$ and $G_0 = 3$. On the left, the relative rate maps show the difference in firing rate in the two cases.

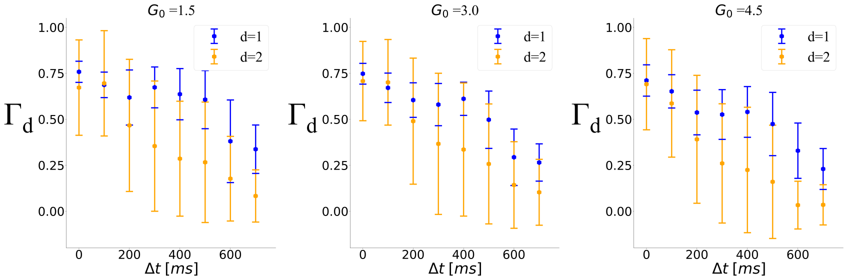

Figure A4: **Dependence of Γ on jitter.** The simulated spike trains with the same parameters as Fig 7 except for the parameters $G_0 = 1.5$, $G_0 = 3$ and $G_0 = 4.5$, are jittered showing the same trend as experimental data.

independent Poisson variability of individual neurons. The way Poisson simulations with oscillations differ from this, and more closely resembles the data, is likely to be the fact that oscillations cause high activity time points to exhibit more regular spiking and less variability in individual neural spiking. In fact, this can be seen at the level of pairwise correlations. While in the real data and simulations with oscillations the average pairwise correlation coefficient is 37% and 27% higher for high activity samples compared to random samples, this increase is only 16% in Poisson without oscillations.

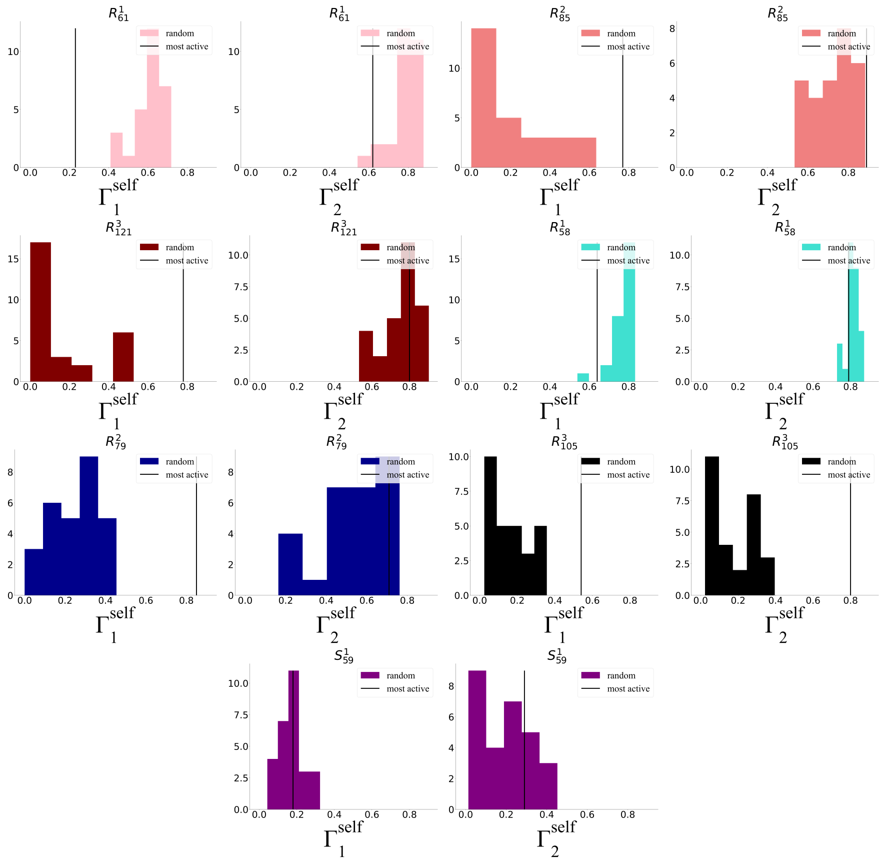

Figure A5: **Persistent homology and the choice of time points in real data.** Each histogram is a set of 30 different random selections of 15000 times for downsampling for the remaining modules. The vertical black line shows the single realization were time points are chosen to be the 15000 ones with highest population vector activity.
