# Supplementary figures and images for: The role of oscillations in grid cells’ toroidal topology
Source: PLoS Comput Biol. 2025 Jan 29;21(1):e1012776. doi: 10.1371/journal.pcbi.1012776 (PMC12165393; doi:10.1371/journal.pcbi.1012776)

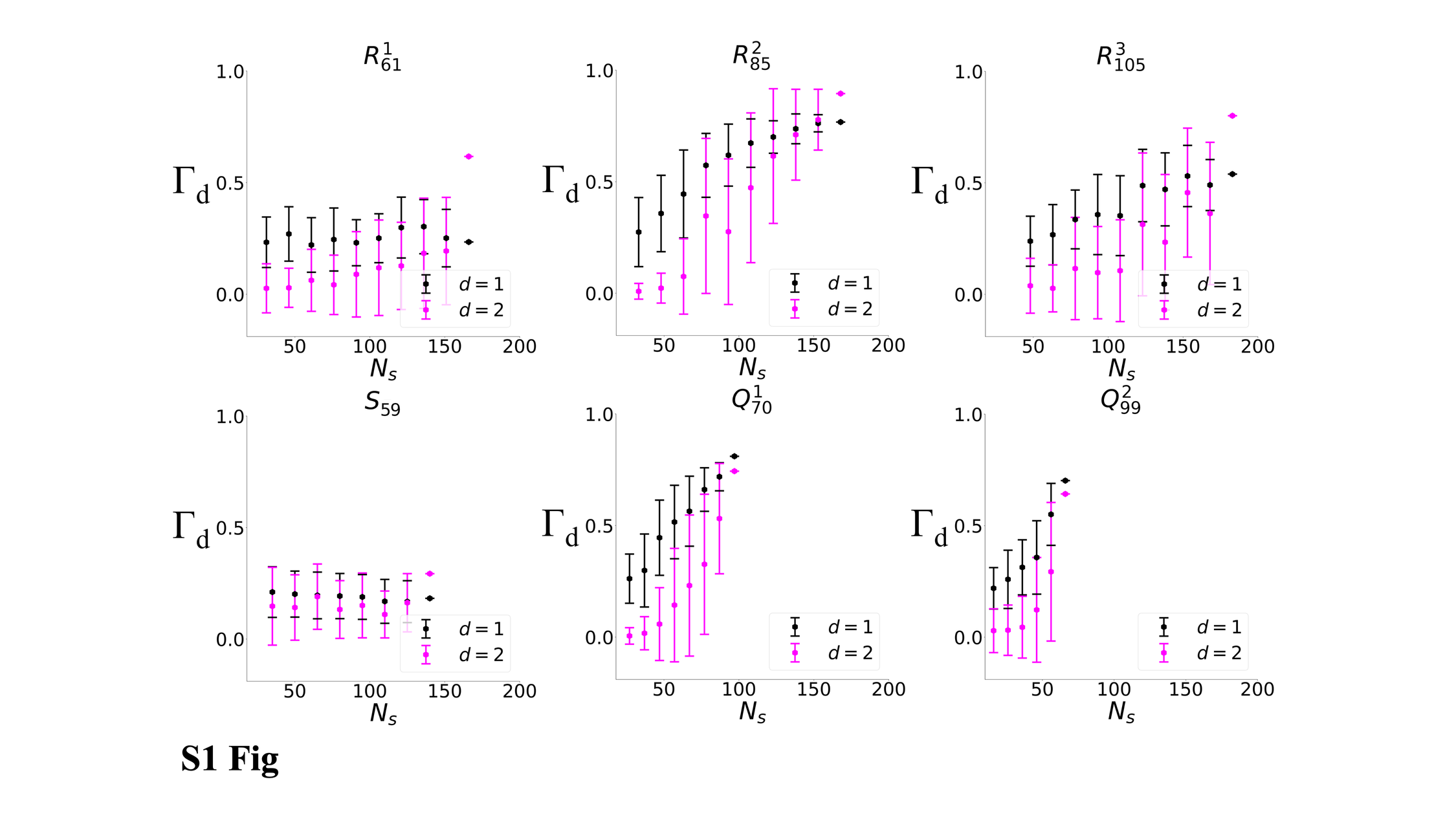

Supplement: S1 Fig — Each plot shows the increase of Γ1 and Γ2 with the number of cells, when all recorded cells are included in the analysis. (TIF) [file pcbi.1012776.s002.tif]

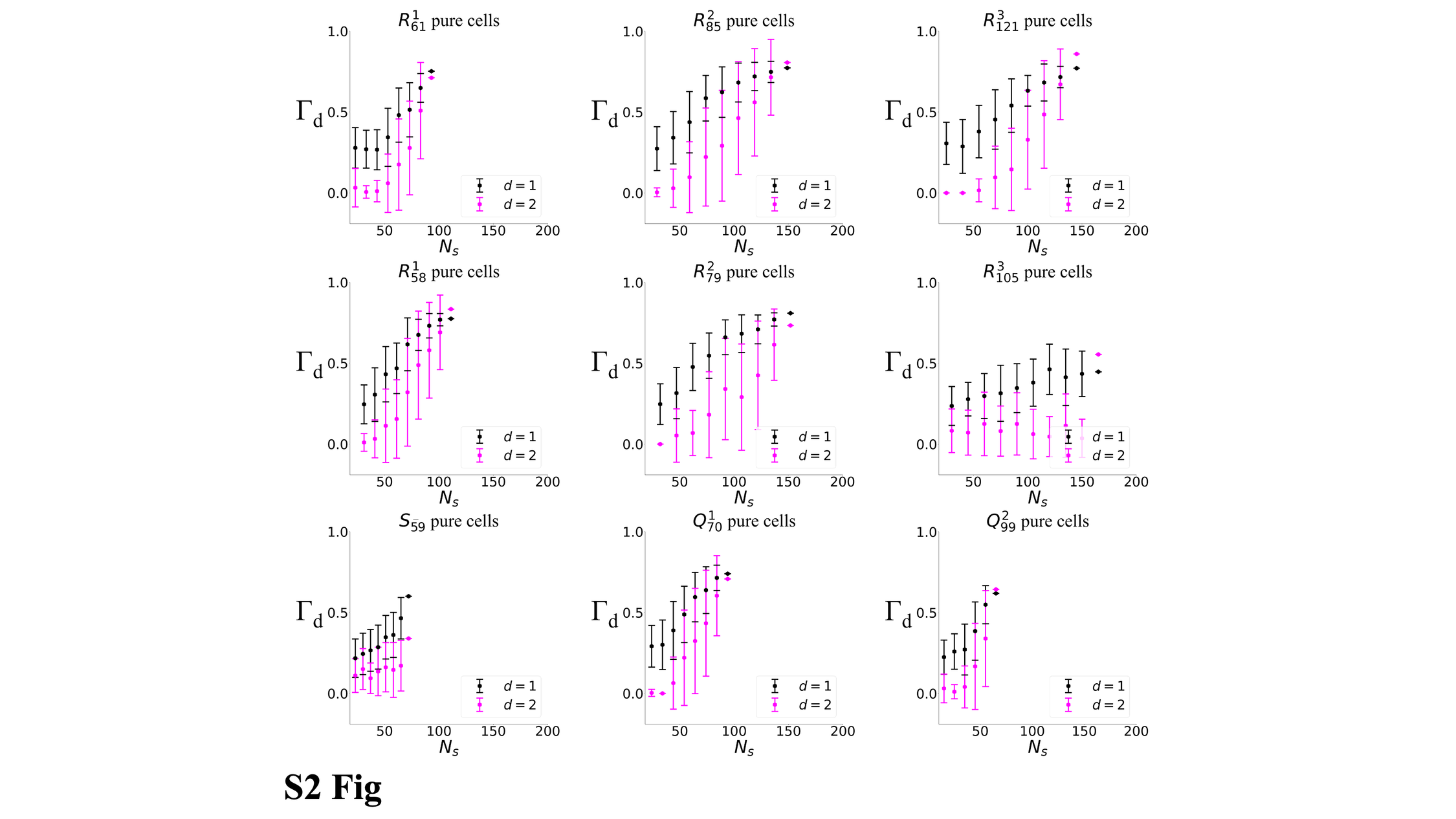

Supplement: S2 Fig — Each plot shows the increase of Γ1 and Γ2 with the number of cells, when pure grid cells only are included in the analysis. (TIF) [file pcbi.1012776.s003.tif]

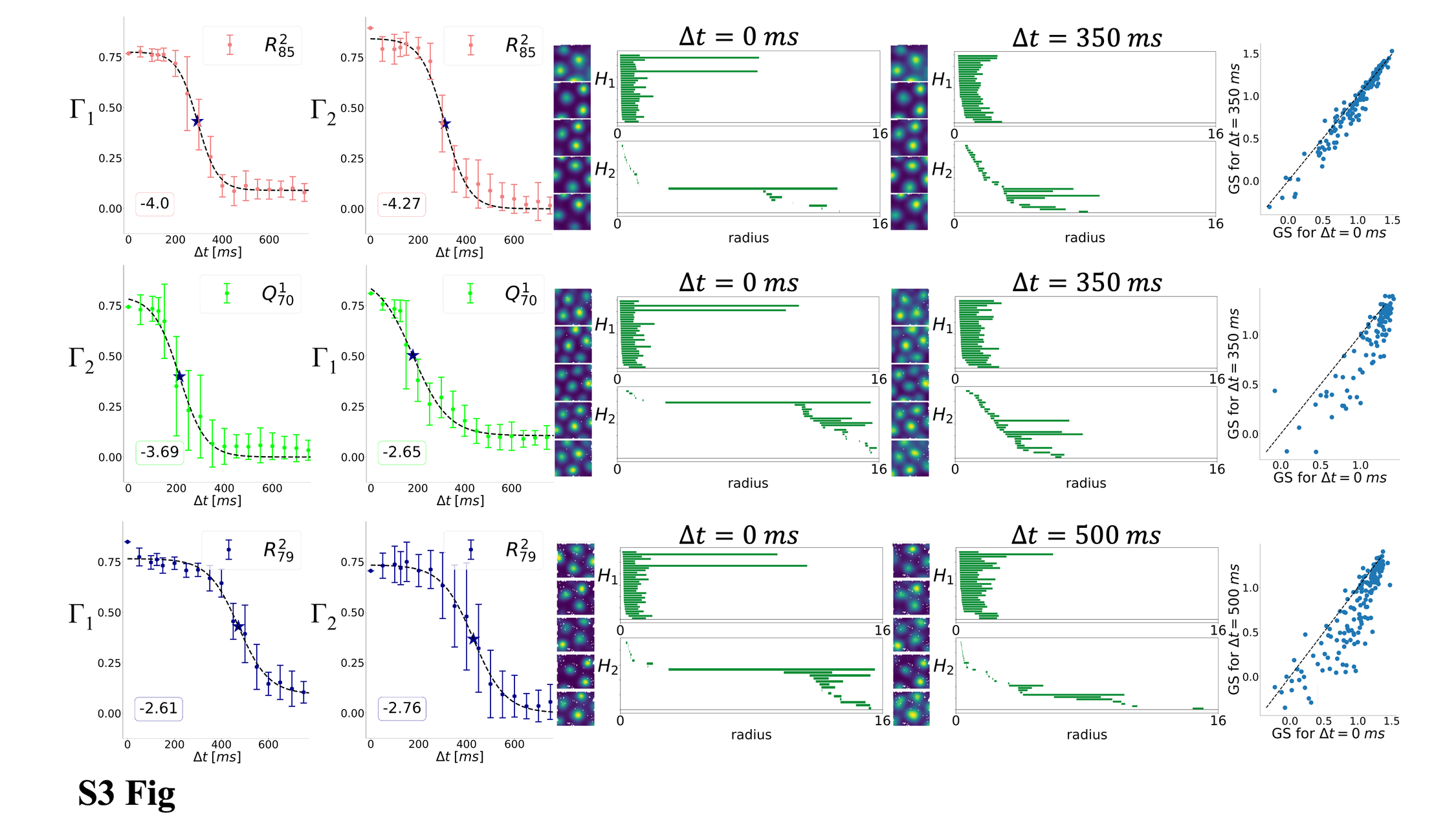

Supplement: S3 Fig — Everything is the same as in Fig 6, but for modules R852, R792 and Q701. (TIF) [file pcbi.1012776.s004.tif]

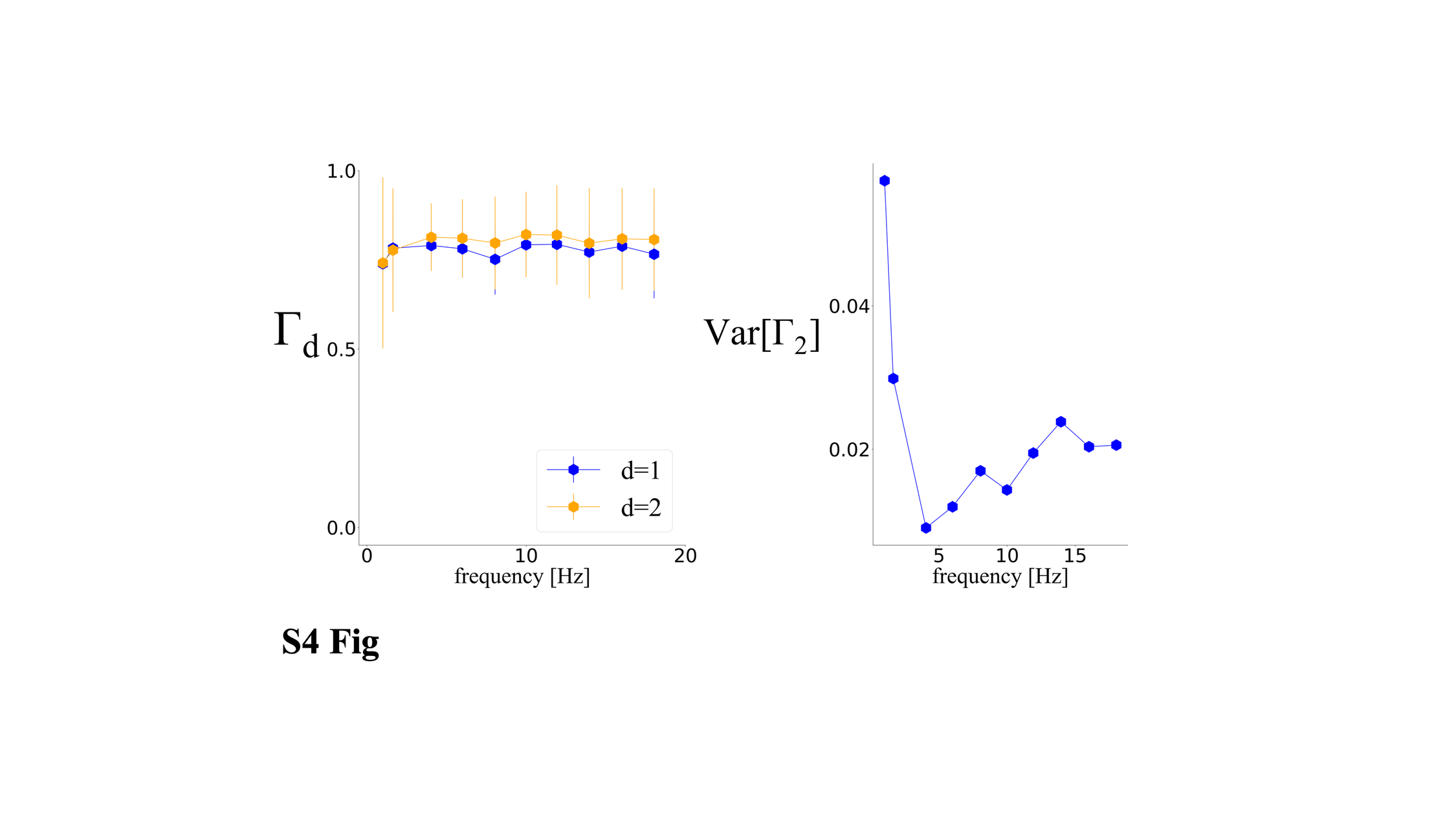

Supplement: S4 Fig — The same simulation as the one in Fig 10A and 10B with smaller spacing (similar to R581) is shown. (TIF) [file pcbi.1012776.s005.tif]

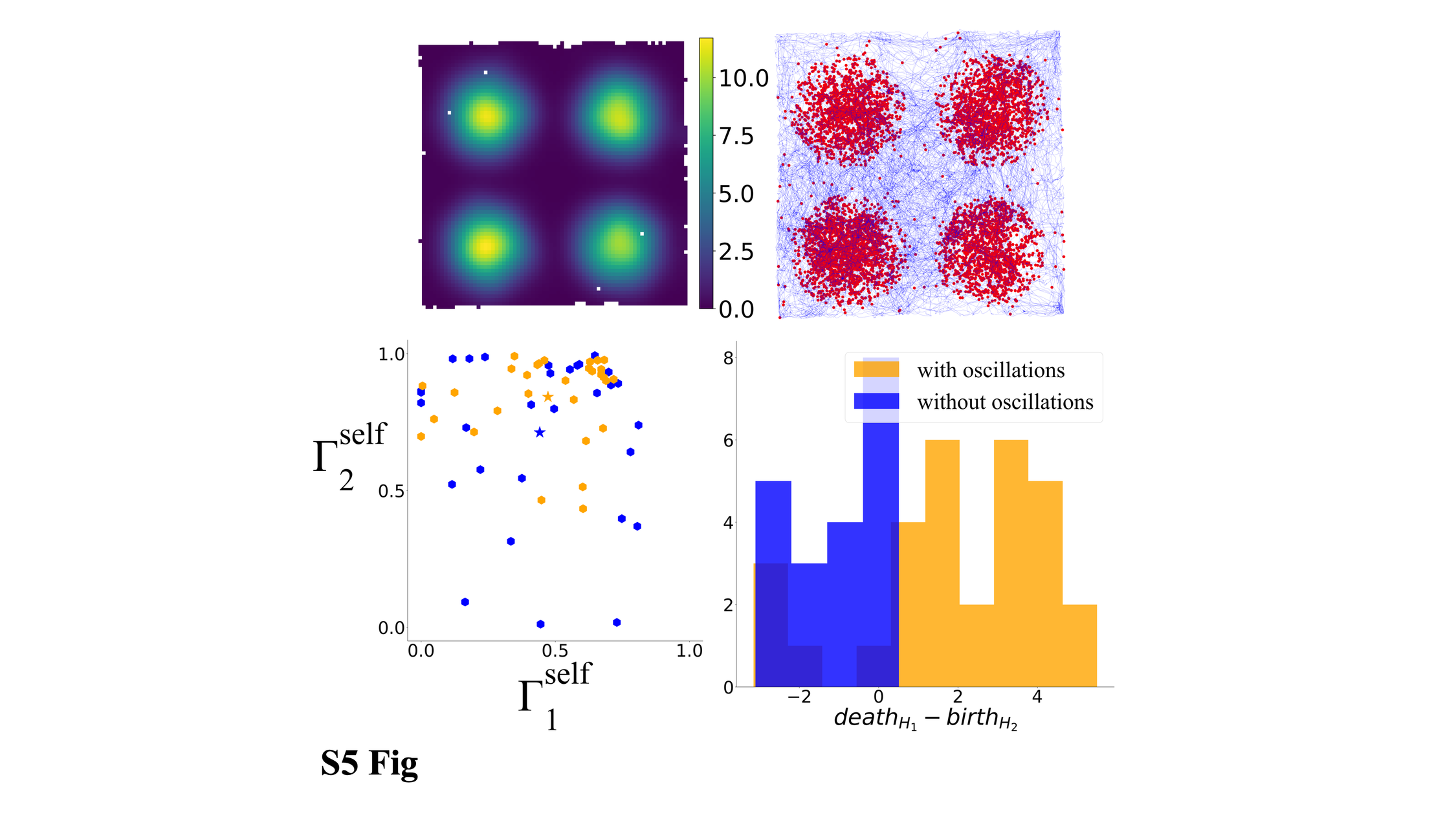

Supplement: S5 Fig — The same simulation as the one in Fig 15 when eta and theta oscillations are introduced for a square grid cell module. (TIF) [file pcbi.1012776.s006.tif]

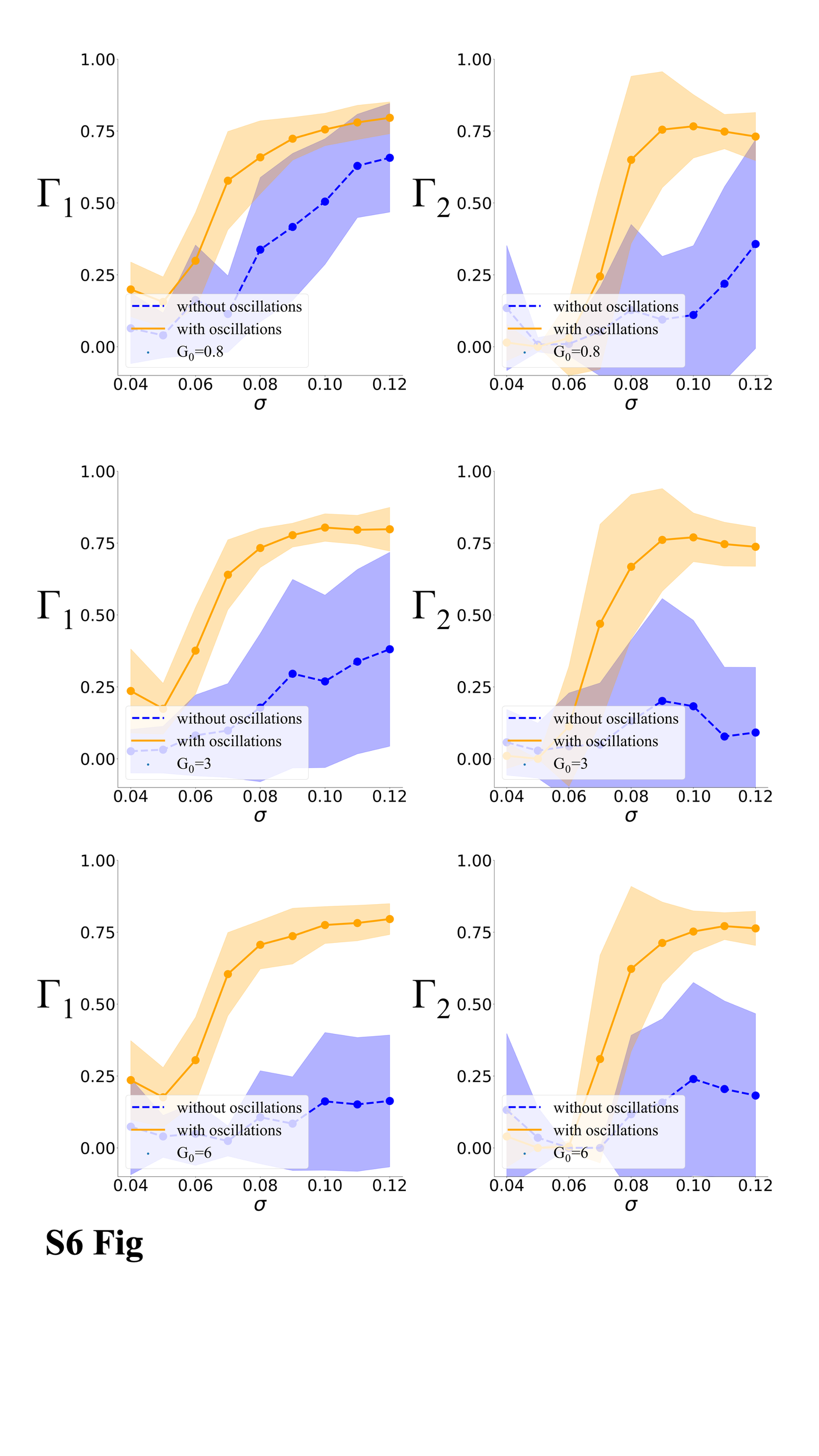

Supplement: S6 Fig — The same simulation as the one in Fig 8 with G0 = 0.8, G0 = 1.5 and G0 = 3 is shown for N = 150. (TIF) [file pcbi.1012776.s007.tif]

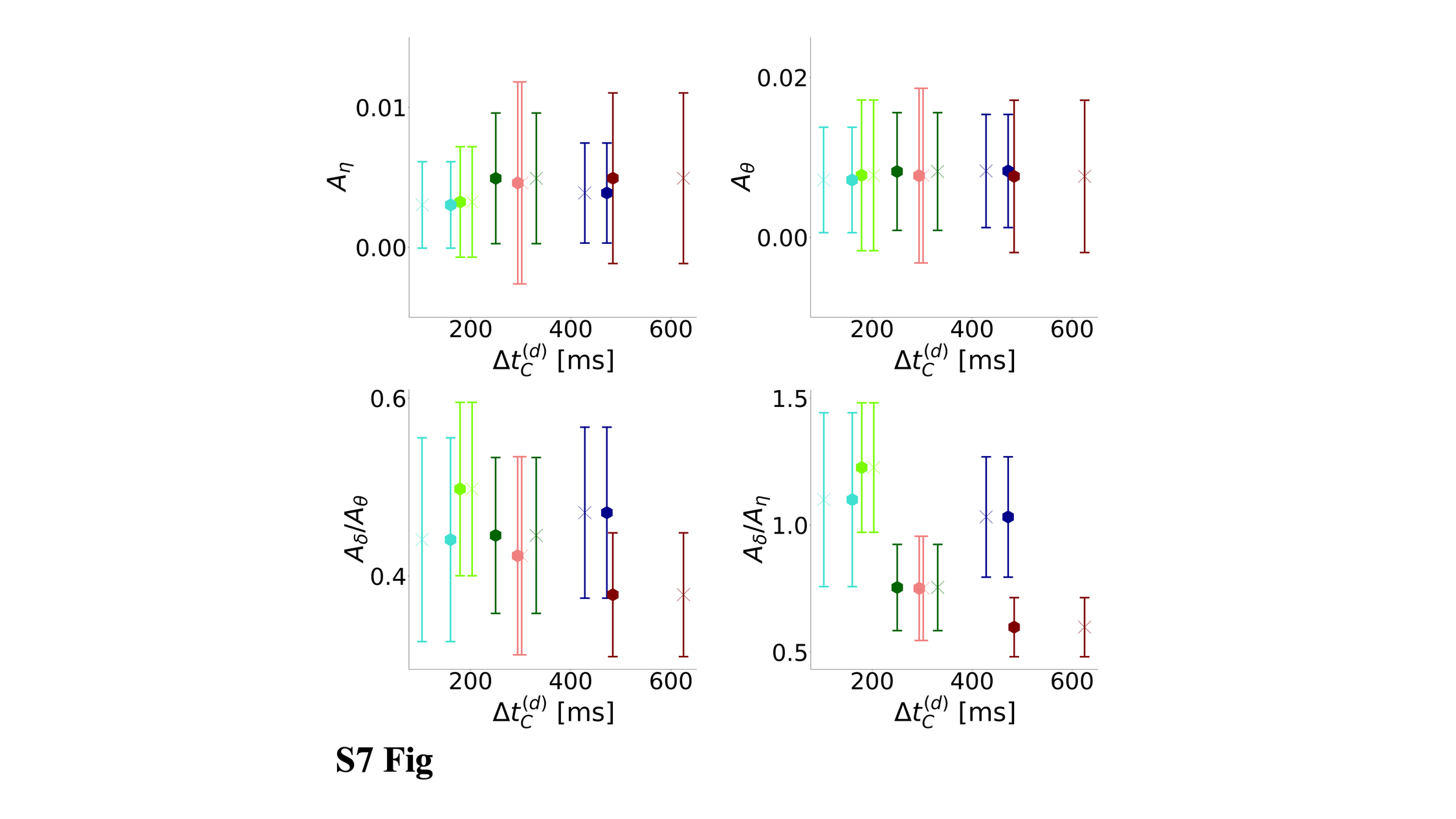

Supplement: S7 Fig — Same as Fig 14 but for Aη and Aθ independently, as well as for Aδ/Aη and Aδ/Aθ. (TIF) [file pcbi.1012776.s008.tif]
