# Supplementary material for: The role of oscillations in grid cells’ toroidal topology
Source: PLoS Comput Biol. 2025 Jan 29;21(1):e1012776. doi: 10.1371/journal.pcbi.1012776 (PMC12165393; doi:10.1371/journal.pcbi.1012776)
Supplement: Attachment — Submitted filename: Coverletter and Reponses.pdf [file pcbi.1012776.s009.pdf]

Authors' Response to Reviews of

The role of oscillations in grid cells' toroidal topology

G. di Sarra, S. Jha and Y. Roudi
PLoS Computational Biology,

RC: ***Reviewers' Comment***, AR: Authors' Response

Dear Editor,

We would like to submit a revised version of our manuscript entitled "The role of oscillations in grid cells' toroidal topology" to PLoS Computational Biology.

We carefully considered each of the comments and made the necessary revisions to the manuscript. In the following, we provide detailed responses to each point raised and outline the changes we have made accordingly.

As suggested by the Editor we have substantially extended our analysis and discussion to include how oscillatory mechanisms compensate for the stochasticity of spiking. We have also made the relevance of our results to CAN models more specific.

We sincerely thank the reviewers for the thoughtful and constructive comments on our manuscript. We appreciate the time and effort put into reviewing our work and providing valuable suggestions to improve its clarity and quality.

Sincerely yours, Yasser Roudi on behalf of the authors.

1. Reviewer #1

RC: ***When considered in neural state space, population responses from a medial entorhinal grid cell module lie on the surface of a torus. This toroidal structure has emerged as an important signature of grid cells. Such toroidal structure naturally emerges from models, but most modeling focuses on rate-based architectures and does not consider the role of spike timing in producing the population structure. In this study, the authors combine experimental data analysis and modeling to show that the synchrony of spikes in the 100-500ms time range is necessary for producing the toroidal topology seen in data. I thought this a conceptually well-organized, well-written and thorough study that convincingly makes the case for thinking about spike timing and oscillations in the grid cell system and that points to the need for richer models. I appreciated the combination of data analysis and modeling and also thought the use of barcode distance was a promising way of probing structure that may find wider use (despite some concerns about the noise sensitivity of barcodes). I did not run the code, but based on a quick glance it seems well-organized and commented (nice job on that!), which greatly increases the chance that future work will build on these analyses.***

AR: We appreciate the review's comments and nice words. We responded to the comments, suggestions, and questions in the following:

RC: ***My comments are primarily aimed at clarifying the description and interpretation of what the authors did. 1) In terms of interpretation, the authors (very nicely) highlight that rate maps of regular fields should give***

rise to toroidal topology but that spiking neurons with irregular fields may not. I particularly appreciated the Poisson simulations, which to me imply that stochastic spiking would be the main cause of the lack of toroidality, and thus that mechanisms to reduce variability in spiking are important to produce toroidality. I had some questions/comments related to clarifying this interpretation:

a) If stochastic spiking is the main issue, would the importance of oscillations and sensitivity to jitter decrease if neurons were to fire at higher rates? A simple simulation showing this in the model would be useful.

AR: We thank the reviewer for raising this issue. To better explore the interplay between the effects of oscillations, spiking variability and firing rate on the detection of toroidal topology we have now ran several new simulations. We have also performed a number of new analyses comparing how the outcome of persistent homology depends on the mean firing rate of the population vectors. The results are shown in Fig 10 and S7 Fig, and described in S1 Appendix as well as added text to the new version, at the end of the section *Computational model of the effect of oscillations* on page 14 . We also carefully discuss these results at the beginning of the new Discussion section.

In the outset, we note that in the TDA approach used by Gardner et al. on real data, and also used by us, only the time bins with highest average population activity are used. In agreement with the reviewer's prediction on the effect of firing rate, our new results show that indeed, in real data, if population vectors are instead from randomly chosen time point, the degree of toroidality is substantially decreased; see S1 Appendix. The same is true for Poisson simulations with oscillations. These choices have the opposite effect in simulations without oscillation; see also Kang et al, ref. 14 in previous draft and the current version. However, simply increasing the average firing rate in Poisson simulations by increasing G_0 in Eq. 4 (newly added) does not change the results; see Fig 10 and S7 Fig. This is largely expected, as for a Poisson distribution, variability in the number of spikes is proportional to the firing rate.

These differences between real data and simulations with oscillations on the one hand, and simulations without oscillations on the other can be explained as follows. In the latter case, high activity population vectors are those in which some neurons generate more spikes above their average rate due to independent Poisson variability. In real data and Poisson with oscillations, the variability is correlated and neurons in the high activity samples exhibit higher correlations. This effect can be seen even when considering pairwise correlation coefficient: in real data/Poisson with oscillations the average correlation coefficient is $37\%/27\%$ higher for high activity samples compared to random samples, while the increase is only 16% in Poisson without oscillations. This is explained in more detail in S1 Appendix.

RC: ***b) Do the Poisson simulations (which have regular firing fields) imply that irregular fields are not an important cause of the lack of toroidality? It would be beneficial to the field if the authors could rule out some mechanisms in addition to pointing to the importance of others.***

AR: We thank the reviewer for the question. We have now performed simulations where the position of each field is increasingly perturbed by independent Gaussian noise. The results are shown in a new figure, Fig 11 of the revised manuscript, and the issue is further discussed in section *Computational model of the effect of oscillations* in particular on page 13 of the current draft.

As can be seen in Fig 11, even with irregular arrangement of the fields in the presence of oscillations (up to to 10%) high toroidality is observed, but then there is sharp degradation in toroidality. These results indeed suggest that in the presence of oscillations, irregularity has a smaller effect on lack of high toroidality than the presence of oscillations.

RC: ***c) Is there an effect of the number of neurons? For example, do the authors predict that modules with***

weak toroidality would show strong toroidality if more neurons were recorded?

AR: We thank the reviewer for this question. To address this comment, we have now added new analyses studying the dependence of toroidality on the number of neurons in the experimental modules; see the new Fig 4 and added text in section *Degree of toroidality of grid cell populations* on page 7 and 8 of the revised manuscript and the newly added S2 Fig and S3 Fig.

These shows that the number of cells definitely lead to higher toroidality in real data, extending the results of Kang et al, including the barcodes in H2. Interestingly, our analysis shows that, generally, Γ varies with N in a much smoother way than with jitter. More importantly, we also find that smaller modules with lower A_η/A_θ exhibit a weaker dependence on N. We can thus predict that in cases with small A_η/A_θ that don't show high toroidality (S_{59} and R^1_{61}), increasing the number of neurons might indeed help, although given the small slope of increase, this likely needs to be a significant increase.

RC: ***2) I was confused as to the reference torus in Fig. 2 and whether it was a good probe for toroidality. As described, the reference torus here involves keeping the two longest bars in H1 and the longest in H2. I see why this might be a good reference to compare jittered data to original data (under the assumption that the original data is toroidal). But it may not be a good way to evaluate the toroidality of the data itself. E.g., say I had data from a plane, calculated its barcodes, kept its two longest bars in H1 and longest bar in H2 (which would all be quite short), and then treated this structure as the reference. In that case I might conclude that the dataset was "toroidal" based on similarity to my reference barcodes. Shouldn't some measure of relative length/persistence of these bars factor into the degree of toroidality of these populations? Relatedly, if the reference is constructed from the experimental data itself, then why is the toroidality of the experimental data not higher? Is this entirely due to differences in shorter bars (that are set to minimum length in reference)? I think I must be missing something about the reference here.***

AR: We thank the reviewer for pointing out the confusion that the definition of the degree of toroidal topology might cause. To clarify this issue we have now significantly revised the section *Defining the Degree of Toroidality* on page 4. We have also added examples in a new figure (Fig. 1), including the case that the reviewer points to (Fig 1 b) to make the procedure clear.

In short, there are two points that we would like to draw the attention of the reviewer to. Firstly, we do not only keep the longest bars. We also set the other bars to have the same starting radius, but with a length set to the bar with minimum length among all bars. This shortest bar may not be visible in the barcodes we show, as the figure only shows the longest 30 barcodes from over 300.

Second, and perhaps more important to address the reviewers comment, relative length and scales of persistence are indeed included in the definition through the normalization we have introduced in Eq.8. This takes care of the situation that the reviewer's example describes as described in Fig 1.

RC: ***3) It would be useful to support the observation that the jitter timescales are "several times smaller than the time it takes for the rat to go from one grid field to another, which is of the order of seconds" given that smearing spikes across firing fields would obviously degrade toroidality. More specifically, given the rats' velocity, what fraction of a firing field width have they moved over the critical timescale of jitters. Does moving locations on the jitter timescale contribute at all to the lack of toroidality?***

AR: We have now added a new section *Estimating time scales* in Methods to describe how we estimate the time it takes for the animal to move from one field to another. For the module R^2_{85}, whose properties were used in most of our simulations, the diameter of the field size for the more regular cells is $\sim 40cm$; see our response to reviewer's comment 8 below. Assuming a running speed of $13 - 15$ cm/sec and straight running, the animal moves $\sim$ 4cm, that is $\sim 10\%$ of the field diameter, at the critical time scale of $\sim$ 300ms for this module.

Having said this, we would like to note that in the dataset we analyzed, field size appears to be an unreliable quantity. In fact, we had attempted to make estimates of field size studying the relationship of field size to A_η/A_θ as well as critical jitter, but we found that estimating field size is highly dependent on the rate map smoothing and affected by the irregular shape of most fields, yielding variabilities larger than the estimated mean field size.

RC: ***4) Sentence on Pg. 8 "Both these indicate that the emerging toroidal topology is much more sensitive to variability in spike times than the smaller ones." The previous lines made me think that the smaller modules were more sensitive but this sentence seems to contradict that. If not a typo it would be good to expand on the point/clarify.***

AR: Thanks for pointing this out. The sentence was indeed a typo. We have now corrected this on page 10.

RC: ***5) Fig. 4: Is there a reason why critical jitter is calculated using Γ_1 for first two modules and Γ_2 for the third?***

AR: We thank the review for pointing this out and revised the caption of Figure 6 and the text on page 10 to clarify this point.

Lack of toroidal topology can either take place because of small Γ_1 (absence of two long bars in H_1) or small Γ_2 (absence of a long bar in H_2). In other words, it is sufficient for the jittering to decrease either Γ_1 or Γ_2 for the toroidal topology to disappear. We have taken the critical time scale to be the smallest time scale at which one of these two quantities significantly decay.

RC: ***6) Eq. 3: Uses the same index for neurons and for temporal frequencies (i). Shouldn't these be different?***

AR: Thanks for pointing this out. In the revised version, we now use μ as the index for the frequencies throughout the manuscript.

RC: ***Could also briefly unpack the sum of oscillation frequencies term in the equation for the reader. In the methods could also describe the normalization of A(omega) and the range and spacing of omegas chosen.***

AR: We have now added further details of the simulation parameters and have included explanatory text in the description of Equation 3 (page 11). We have also extended the section *Parameters of the oscillations* in Methods to include more details on the oscillation frequencies and amplitudes.

RC: ***7) What are the values of firing rates for simulations in the center of a firing field and how do they compare to data? Relatedly, it would be useful to add a scale to the firing rate maps throughout, including in the simulations (especially if the effect is dependent on firing rate).***

AR: We thank the reviewers for this question. For the simulations in Figure 7,8 and 9, the firing rate of each cell in the center of the field is around 13-16 Hz. This is shown in Figure 7, where we have now included the color-code for the relative rate maps. In simulations, average firing rates and peak firing rates are determined by G_0 and σ in Eq. 3 and 4. We have chosen the value of these parameters so that the average number of spikes over all neurons is similar to that of real data and the field sizes are comparable; see also our responses to your comment 8 below. This was done because the peak firing rate in the fields in real data is heavily dependent on smoothing, the variability from field to field is very significant (e.g. from a few hertz to over 30Hz); see also Dunn et al. ref 29 and Ismakov et al. 31. In response to this comment as well as reviewer's comment 1, we now also show how varying G_0 influences our results (Fig 10, S7 Fig and S1 Appendix). The results show that the difference caused by the addition of oscillations is present over a considerable range of G_0.

RC: ***8) Fig. 6: what is the value of sigma that best fits the experimental data? Is the best match between simulations and data at this value of sigma, or does it continue to increase with bigger sigma? If it does continue to increase, why is this?***

AR: Setting σ in the range $\sigma = 0.1 - 0.12$, generates field sizes that resembles real data for the module spacing we consider in the simulations (R^2_{85}); see the figure below.

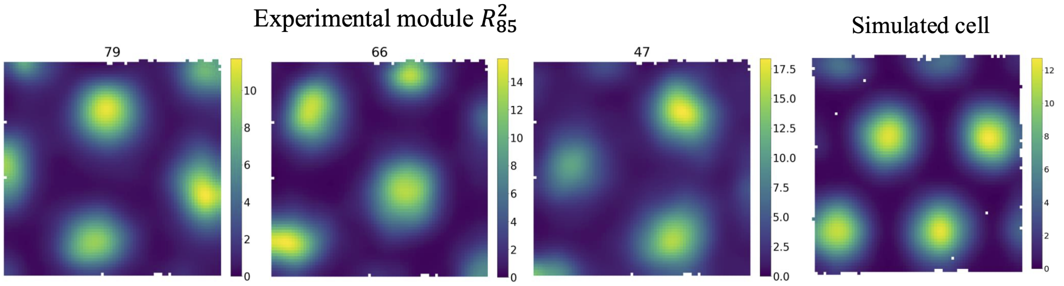

Figure 1: **Comparison between experimental and simulated grid cells.** Grid cells from module R^2_{85} are shown on the left. The identity of each cell is shown on the top of each plot. One example of simulated grid cell is shown on the right. A simulated module is composed of identical cells where the hexagonal grid is shifted.

As mentioned above in comment 3, however, estimating quantitatively the field size is unreliable: it is highly dependent on the rate map smoothing and affected by the irregular shape of most fields.

RC: ***9) I found the results on eta-to-theta ratio somewhat confusing. The authors show in simulations and figures that including oscillations in these frequency bands improves toroidality. However, these results seem disconnected from results on the ratio of eta to theta power. As far as I can tell, Fig. 11 is showing that including these oscillations improves toroidality but not showing that these oscillations need to have a high ratio or modulating the ratio. It was also not clear why mechanistically the ratio of these oscillations should be the important feature for toroidality (rather than simply oscillations in these power bounds). The Discussion seems to hint that these results are being driven by changes in the eta power (which would then change the eta to theta ratio, assuming that theta did not also vary similarly in a way that kept the ratio the same). If so that could be made more explicit and might be a simpler framing.***

AR: Thanks for this comment. We have now made the transition from the section on simulations results to that of the experimental analysis of eta-to-theta power smoother by adding a new paragraph at the end of the former section.

The main point is that real data oscillations are correlated with other features of the grid cells (module spacing, field size etc) while in the model, oscillations can be independently set. As such we used simulations only to confirm the basic hypothesis that we made based on the jittering results: that there is a role for oscillations in the eta and theta bands in the appearance of toroidality. In fact, in response to comment 1 of Reviewer 2, we now show that even the presence of a single oscillation at different bands does increase toroidality in simulations, although it does so more robustly in the eta-theta band and their harmonics. Given the complex role that oscillations play in relation to various features of grid cells, to understand precisely what aspects of theta and eta quantitatively correlate with temporal jitter, we thus directly looked at the real data. We have now added a paragraph to the end of *Computational Model of the Effect of Oscillations*, page 14 to make this clear.

RC: ***10) For completeness, some more information on how the TDA was performed could be included in methods (I realize that the authors follow Gardner et al.). For example, the PCA preprocessing step is briefly mentioned in main text but not in Methods.***

AR: Thanks for the suggestion. Indeed since the TDA procedures are varied and involve a number of steps, it is helpful to include a summary. We have thus extended the section *Experimental procedures and preprocessing* in Methods (page 21), to add more details on the preprocessing steps, and included it in the Introduction (see our resonse to point 1b above). S1 Appendix further focuses on the effect of preprocessing. This issue is also mentioned in the Discussion on page 20.

2. Reviewer #2

RC: ***In thsi manuscript, di Sarra et al. present a novel framework to understand the relationship between grid cell spatial patterns of activity and the description of such activity as living on a toroidal manifold. The main claim of the paper is that these two properties associated with grid cells are mostly independent, being based on the organization of activity over largely separated time-scales (seconds vs. 100-400ms). In particular the authors claim that the emergence of a toroidal topology explaining the relative configuration of population activity patterns can be ascribed to the modulatory effects on average activity of oscillations in the thera and eta band of the spectrum. When spiking activity is subject to the combination of thse two oscillations, toroidaility is found to be strengthened in a model of grid cell activity, while a strong modulation in these two bands in experimental data is associated with stronger evidence of a toroidal topology. Importantly, hexagonal arrangment of fields seem to be mostly a separated property. In my opinion the authors present two main contributions. The first one is to define a measure of proximity to a toroidal topology. The toroidal hypothesis is gathering momentum in the study of grid cells, and with it the reliance on topological methods to define the properties of this neural population. Most of this evidence, though, is based on a significance-based assessment, that is a zero or one measure of compatibility between the neural activity and a toroidal arrangment of the activity patterns. Such dichotomous approach risks to mask the variability in the arrangement of activity and to lead to an oversimplification or a distortion of the actual richness in activity structures. Therefire I find the introduction of the similarity measure quite important as it allows to capture the proximity and the degree of proximity to a torus, providing information on the appropriateness of the description. Such measure also allows to more precisely measure the contribution of specific features of neural activity in driving the system towards or away from toroidailtiy. Accordingly, the second contribution of the paper, is to explicitely investigate which aspects of neural activity can be identified as most relevant for approaching a toroidal topology. The separation between 'spatial coding' (in this case the hexagonality of the field arrangment) and 'temporal coding' (the modulation of activity at timescales generally shorter than the behavioral one, in most of the cases involving theta oscillations) is quite popular when it comes to the study of the hippocampus and neighboring cortices, but it has never been applied to this problem. The results of the paper are in many senses surprising, as they seem to dissociate the presence of hexagonal firing and that of torodaility. The analysis seems to be still mostly at the qualitative level, as there is no precise mechanism proposed in the paper to explain the role of oscillations. At the same time both the experimental and the model analysis are quite convincing in making the case for a prominent role of oscillations in the emergence of toroidaility. To fully appreciate the relevance of the approach and of the model though, I think that a number of additional analyses would be helpful.***

AR: We thank the reviewer for the thoughtful and nice words. In the following, we performed the requested additional analyses.

RC: ***1. My first point regards the role of oscillations: increased coherence in spikes is mentioned as their role in shaping the torus, but why 2 oscillations? Does it work only with one modulated band? And is there anythig specific about these two bands? It would be very useful to further contrast the behavior of the model by using only one of the two oscillations and by changing their frequency.***

AR: We thank the reviewer for the comments and questions. We had built the model with two dominant oscillations, one at eta and one at theta, due to the biological plausibility of the the presence of oscillations in these bands. Following the reviewer's comment, we have now performed the suggested analysis, keeping just one dominant oscillation band, and varying its frequency between 1 and 20 Hz. This analysis is shown in Fig 10 and S5 Fig.

The new analysis demonstrates that, in general, even one oscillator is enough to yield a high degree of

toroidality in the simulation. Importantly, however, we find that how robustly one can get high toroidal does depend on the frequency chosen. This is reflected in the fact that the variability of Γ_2 from one simulation to another shows a minimum in the eta to theta range and their harmonics.

RC: ***2. Hexagonal arrangement is shown to be not sufficient for having a torus. But is it necessary? It would be greatly informative to run simulations replacing grid cells with units having multiple fileds spanning the environment and mantaing a similar distance from each other, but lacking the precise arrangement of an hexagonal tesselation.***

AR: We thank the reviewer for the comments. We performed simulations replacing grid cells with cells showing a square grid, keeping a regular non-hexagonal organization. To show that the effect of oscillations is not dependent on the hexagonality of the grid, we estimate $\mathbf{\Gamma}^{self}$ in the cases with and without oscillations. The simulation is performed in the exact same way as for the hexagonal case and it is shown in S6 Fig, referred to at the end of *Computational model of the effect of oscillations* on page 13 of the revised manuscript; barcodes consistent with toroidal topology are present in this case. Furthermore, even when hexagonal organization is degraded by randomly moving the field locations by up to 10-15 % of the grid spacing, toroidal topology is still detectable; see Fig. 11 of the revised manuscript. As can be seen at 8-10 % displacement, the hexagonality is hardly visible in the rate maps. These results show that the hexagonal arrangement of the fields is not necessary.

RC: ***Parallely it would also be interensting to test the degree of torodality in the non-grid population in the dataset. Figure 2 seems to suggest that the inclusion of non-grid cells is not affecting the torus, and if anything is increasing the degree of torodality. This would indicate that each of the populations has an high degree of torodality.***

AR: We would like to note that the dataset does not include non-grid cells as such, only grid cells without head directional tuning (Pure Grid cells) and with head directional tuning (Grid-HD). We have made a more careful analysis of the effect of Grid-HD cells in a new panel added to Figure 3 and we refer to it in *Degree of toroidality of grid cell populations* on page 6. This shows the relative change in toroidality between pure cells only and all cells.

It is to be noted that in most cases the toroidality change is close to zero, in one case (R^3_{105}) toroidality increases by considering all cells, and in two it decreases (S_{59} and R^1_{61}). Non-pure grid cells may decrease toroidality because of their selectivity to other variables (e.g. head-direction), that would create a topological circle. However, they still show hexagonal tuning, so they could contribute to increase toroidal topology. This is also reasonable to assume, because they significantly increase the total number of cells in the analysis. Testing toroidality on non-pure grid cells only would be unpractical, since their number is only a small fraction of total cells.

RC: ***3. Putting these two points together, I also wonder wether higher degree in small grids could be achieved by using higher frequency ranges, allowing for a better separation between temporal activity and the behavioral scale of field crossing.***

AR: We thank the reviewer for the question. We performed the same simulation as point 1 for smaller spacing and shown the results in S5 Fig. This figure shows a similar dependence on the frequency when compared the medium module size in Fig. 10.

3. Reviewer #3

RC: ***In the submission "The role of oscillations in grid cells' toroidal topology" the authors re-analyze data from Gardener et al. 2020 and, by using simulations, investigate possible contributions of oscillations to the toroidal topology. In this manuscript, a metric for the degree of toroidality based on persistence homology is introduced, and temporal jitters, together with simulations, are used to assess the robustness of toroidal topology. The manuscript is timely, and it's topic of potential interest and within the scope of the Journal.***

AR: We thank the reviewer for the appreciation towards our work.

RC: ***Although the motivation and writing of the paper are clear, the methods are not always described clearly enough to fully assess the validity of the simulations and, therefore, conclusions drawn from them.***

AR: We appreciate the reviewers' comments and suggestions and have made substantial changes to the manuscript to make it more clear. In particular, we have now expanded the methods section describing the simulation in more details. Please see our responses and specific changes we have made below.

RC: ***The results presented in the manuscript suggest a potential contribution of oscillations to toroidal stability. However, while discussing CAN models versus weighted feedforward, input-driven networks, it is less clear how these results favor one or the other. The two points made on the bottom of page 16, could be made without any of the results described in the manuscript. The fact that an alternative model leads to similar toroidal topography demonstrates a possibility but does not rule out CAN models. As later addressed in the manuscript, attractor dynamics do play an essential role in grid cell populations. Therefore, the above-mentioned comparison of models needs either to be toned down or seamlessly related to the manuscript's results. Please find my specific comments below.***

AR: We agree that the majority of the criticism of the Path-Integrating CAN model is independent of our results. We have now made it more clear how our results add to the discussion around Path-Integrating CAN models and feed-forward models for the generation of hexagonal patterns, as well as continuous attractor dynamics in general. We also now separate the Summary of our results from that of the Discussion and make it clear that disentangling the effects of Path-Intergation, CAN dynamics (with or without Path-Integration), recurrent connectivity, and oscillations cannot be conclusively made based on our results, but requires further word. We also discuss possible approaches.

Major comments

RC: ***1.) Parameters of the simulations should be stated more clearly, both in the results section (page 4), and in the methods sections; at least i.) how many neurons were simulated, ii.) how many realizations with what parameters, iii.) whether the oscillations in eq. 3 were synchronous for all neurons simulated.***

AR: We thank the reviewer for pointing this out. We have added more details in the result section, specifying the number of neurons, the number of realizations for each simulation and the features of oscillations on page 11. We also extended the paragraph *Parameters of the oscillations* in Methods.

RC: ***2.) Although it seems to be intended in the simulations, one could argue that the oscillatory part in eq. 3 limits the temporal variability of neuronal firing, increasing correlation within the simulated population and hence facilitating toroidal topology, given the presence of repetitive firing fields. The key question is whether any other mechanism, e.g., organized, sequential firing, that limits the temporal variability between neurons would not have the same effect.***

AR: We thank the reviewer for the suggestion. As the reviewer correctly points out, other mechanisms could limit temporal variability and increase correlations necessary for the detection of toroidal topology. We have now added these other possibilities in the Discussion (page 20) but we think exploring the effect of all these other factors are beyond the scope of one paper. In particular, we believe a careful analyses of these factors would require analyzing the timing of spikes with respect to LFP signal that we do not have access to (see response to comment 5). We also suggest that some insight into the effect of other mechanisms can gained by focusing the analysis on selected time points (see also our point to your comment 3 below).

RC: ***3.) The sampling of binned spike counts needs to be stated explicitly; Gardner et al. 2022 used 250ms intervals between samples, which would be in the range of the critical jitter times described in the manuscript. It would be relevant to rule out whether this binning had any influence on the results reported.***

AR: We thank the reviewer for the suggestion. The 250 ms sampling of binned spike counts in Gardner et al. relates to the UMAP visualization (see *Visualization of toroidal manifold* in Gardner et al.), but not to the topological data analysis. In fact, the down-sampling for TDA consists of sorting the time bins according to their mean activity in the population vectors, and selecting the 15000 most active time bins.

To further understand how this choice of sampled time point influences the outcome of persistence homology (which is also related to comment 1 of Review 1), we now explore and discuss this issue in detail in page 14 of the revised version of the manuscript and a new supplemental material S1 Appendix. We show that selecting the time points randomly, significantly degrades the toroidal topology in real data and in simulations with oscillations but has an opposite effect of Poisson simulations in the absence of oscillations. We discuss the implications of these findings in the Discussion and S1 Appendix.

RC: ***4.) On page 3, there is mention that the absolute power of theta or eta power did not correlate with critical time scales, but these results are not included in the manuscript, nor is whether the relative power of eta or theta (to population average) might play a key role.***

AR: We thank the reviewer for pointing this out. What we meant was that A_η and A_θ did not individually correlate with the critical jitter size. We have now added S8 Fig showing this.

RC: ***5.) Given the proposed relevance of oscillations, does the amount of i.) theta-modulated cells or ii) cells showing phase-precession impact toroidal topology?***

AR: We appreciate the interesting suggestion. Unfortunately, the data set available online at `https://figshare.com/articles/dataset/Toroidal_topology_of_population_activity_in_grid_cells/16764508?file=35078602`, that is the one analyzed in this paper, only contains the spike times for each recorded grid cell. Thus, connecting the dynamics of the LFP to spiking activity is not possible with this kind of data. However, we mentioned the importance of the topic, including possible difficulties in estimating it in open environments in the Discussion on page 20.

RC: ***6.) The numbers in Table 2 might be incomplete; from my understanding, module spacing and critical jitter times should be listed here.***

AR: We thank the reviewer for asking. The spacings are reported in Table 1. Critical jitter time is reported in Table 2, along with the name of each module, where the subscript indicates the module spacing. Since module $S_5 9$ doesn't show strong toroidality, it doesn't have a critical time scale, and so it is thus not included in Table 2 of the current version.

RC: ***7.) The manuscript sometimes reads like a commentary on Gardner et al. 2022, especially on page 6 when discussing the results of Extended Fig. 6A or when commenting on the Wagon Wheel environments***

on page 16. Usually, present results are discussed in the context of previous literature. I would suggest limiting these comments or discussions to the relevance of the present results of the submission.

AR: We appreciate the reviewer's comment. We have gone through the text and revised the paper so as to address this issue. In particular, we have changed the text referred to by the reviewer and significantly revised the Discussion making our comments and discussion more directed towards our own results.

3.1. Minor comments

RC: ***A.) The second to last sentence of the abstract is hard to understand. Consider re-writing or splitting it into two sentences.***

AR: We agree that the sentence was not clear enough and we rewrote it. Thanks!

RC: ***B.) The introduction presents not only the context and motivation of the study but also partly discusses the results, which I found unusual for an introduction. I would consider limiting to context, motivation, and a brief outline of the study results.***

AR: Given the reviewer's comments as well as changes made throughout the manuscript in response to all the comments, we have substantially revised the introduction. We now limit it to the context and motivation and only briefly outline the results.

RC: ***C.) Given the similarity in Fig.1 a and e, why is the x-axis in e) not matching the one in a) ?***

AR: Thanks for pointing this out. The parameters of this simulations are reported in *Methods* on page 23. Given the smaller size of the torus in 6D, compared to the ones in 3D, weaker noise is sufficient to reduce toroidality.

RC: ***D.) For a better illustration of the comparisons following Fig. 2, would a difference plot (all minus pure grid cells) help to show the relative in-/decrease of tau parameters in 2D?***

AR: We thank the reviewer for this suggestion. We introduced a new panel to Fig. 3 in the current revision (Fig 2 of the previous version) where the relative difference (difference of $\Gamma_{1,2}$ divided by the sum) is shown and discussed on page 6.

RC: ***E.) It would be helpful to add the integration time constant and time between grid fields on page 8, such as in the discussion (pg.15).***

AR: We thank the reviewer for the question. We introduced a paragraph estimating the behavioral time scale in *Estimating time scales* in Methods page 24, and referred to it in the main text.

RC: ***F.) From my understanding, the peaks shown in Fig. 5 at 4 and 8 Hz in the PSD only show that the simulation worked. As of now, it reads like a result; it would be surprising if we did not see peaks here, given the oscillations included in the simulations. Please clarify or mention these are peaks confirming the functionality of the simulations.***

AR: The PSD in Fig 5 of the previous manuscript version, and Fig 7 in the current one, indeed only shows the shape of the PSD that is put into the simulations and is not meant to be a result. We have modified the text and rephrased the caption in Fig 7 to clarify this and avoid confusion.

RC: ***G.) Is the correlation mentioned in Fig. 10b computed from N=6 values? Please state explicitly in the caption.***

AR: We thank the reviewer for noticing. We rewrote the Figure 14 caption of the current version (Fig 10 of the

previous) to make the point clearer.
